# HerzMobil, an Integrated and Collaborative Telemonitoring-Based Disease Management Program for Patients With Heart Failure: A Feasibility Study Paving the Way to Routine Care

**DOI:** 10.2196/cardio.9936

**Published:** 2018-04-30

**Authors:** Elske Ammenwerth, Robert Modre-Osprian, Bettina Fetz, Susanne Gstrein, Susanne Krestan, Jakob Dörler, Peter Kastner, Stefan Welte, Clemens Rissbacher, Gerhard Pölzl

**Affiliations:** ^1^ Institute of Medical Informatics UMIT – University for Health Sciences, Medical Informatics and Technology Hall in Tirol Austria; ^2^ Center for Health & Bioresources AIT Austrian Institute of Technology Graz Austria; ^3^ Tyrolean Health Care Fund Innsbruck Austria; ^4^ Clinical Division of Cardiology and Angiology Medical University of Innsbruck Innsbruck Austria; ^5^ Center for Health & Bioresources AIT Austrian Institute of Technology Hall in Tirol Austria

**Keywords:** heart failure, telemedicine, delivery of health care, integrated, program evaluation

## Abstract

**Background:**

Heart failure is a major health problem associated with frequent hospital admissions. HerzMobil Tirol is a multidisciplinary postdischarge disease management program for heart failure patients to improve quality of life, prevent readmission, and reduce mortality and health care costs. It uses a telemonitoring system that is incorporated into a network of specialized heart failure nurses, physicians, and hospitals. Patients are equipped with a mobile phone, a weighing scale, and a blood pressure and heart rate monitor for daily acquisition and transmission of data on blood pressure, heart rate, weight, well-being, and drug intake. These data are transmitted daily and regularly reviewed by the network team. In addition, patients are scheduled for 3 visits with the network physician and 2 visits with the heart failure nurse within 3 months after hospitalization for acute heart failure.

**Objective:**

The objectives of this study were to evaluate the feasibility of HerzMobil Tirol by analyzing changes in health status as well as patients’ self-care behavior and satisfaction and to derive recommendations for implementing a telemonitoring-based interdisciplinary disease management program for heart failure in everyday clinical practice.

**Methods:**

In this prospective, pilot, single-arm study including 35 elderly patients, the feasibility of HerzMobil Tirol was assessed by analyzing changes in health status (via Kansas City Cardiomyopathy Questionnaire, KCCQ), patients’ self-care behavior (via European Heart Failure Self-Care Behavior Scale, revised into a 9-item scale, EHFScB-9), and user satisfaction (via Delone and McLean System Success Model).

**Results:**

A total of 43 patients joined the HerzMobil Tirol program, and of these, 35 patients completed it. The mean age of participants was 67 years (range: 43-86 years). Health status (KCCQ, range: 0-100) improved from 46.2 to 69.8 after 3 months. Self-care behavior (EHFScB-9, possible range: 9-22) after 3 months was 13.2. Patient satisfaction in all dimensions was 86% or higher. Lessons learned for the rollout of HerzMobil Tirol comprise a definite time schedule for interventions, solid network structures with clear process definition, a network coordinator, and specially trained heart failure nurses.

**Conclusions:**

On the basis of the positive evaluation results, HerzMobil Tirol has been officially introduced in the province of Tyrol in July 2017. It is, therefore, the first regular financed telehealth care program in Austria.

## Introduction

### Heart Failure as a Major Public Health Problem

Heart failure is approaching epidemic proportions worldwide and represents a major public health problem. Prevalence of heart failure is estimated to be 1% to 2% of the adult population, rising to 10% and more in persons aged 70 years and older [1,2]. Heart failure is the leading cause of hospitalization among elderly patients [3]. Rates of death and readmission remain high despite considerable advances in medical therapy. Mortality rate within the first year approaches 40%, and up to 50% of patients are readmitted within 6 months of discharge [4-6]. Repeat hospitalizations are associated with higher mortality and contribute substantially to the enormous overall economic burden of the disease [7]. The risk of readmission and death is greatest in the early period after discharge [7-9]. These findings suggest a role for increased surveillance in the early postdischarge period of greatest vulnerability after heart failure admission. It is estimated that up to two-thirds of readmissions for acute heart failure are triggered by potentially remediable factors including “poor discharge planning, nonadherence to recommendations regarding diet and medical treatment, inadequate follow-up, poor social supports, and delays in seeking medical attention” [10].

### Heart Failure Disease Management Programs

Multidisciplinary postdischarge disease management programs have been established to prevent readmission and to reduce mortality and health care costs. A recent systematic review of 47 trials took into account the heterogeneity in models of care used in different studies: multiprofessional heart failure clinics, multiprofessional follow-up without heart failure clinics, telephone contact, primary care follow-up, and enhanced patient self-care [11]. In this review, home-visiting programs and clinic-based multidisciplinary programs reduced all-cause readmission within 3 to 6 months by 25% and 30%, respectively. Comparable results have also been reported in elderly patients [12,13]. Interestingly, a more recent review published by Ong et al showed no significant differences in 30-day readmission rate or 180-day mortality, but a significant difference in 180-day quality of life between the intervention group comprising nursing-lead phone calls and telemonitoring and the usual care group [14].

On the basis of this evidence, the European Society of Cardiology recommends heart failure care delivered in a multidisciplinary program with high priority [15]. The fundamental role of heart failure nurses is particularly emphasized [16]. In addition, a recent position paper of the Austrian Society of Cardiology recommends multidisciplinary disease management programs with monitoring of high-risk heart failure patients [17]. Exact workflow definition and the specific role of participating stakeholders in a multidisciplinary telemonitoring-based disease management program, however, still have to be defined.

HerzMobil Tirol is such a multidisciplinary disease management program for heart failure care. It was developed and implemented in the Austrian province of Tyrol.

### Objectives

The objectives of this study were to evaluate the feasibility of HerzMobil Tirol by analyzing changes in health status as well as patients’ self-care behavior and satisfaction and to derive recommendations for implementing a telemonitoring-based interdisciplinary disease management program for heart failure in everyday clinical practice.

## Methods

### HerzMobil Tirol

HerzMobil Tirol is a multidimensional postdischarge disease management program for heart failure patients using a telemedical monitoring system incorporated in a comprehensive network of specialized heart failure nurses, private practice physicians, and 3 secondary and 1 tertiary referral centers. The aim of HerzMobil Tirol is to achieve relevant and stable impact on readmission rates, mortality, quality of life, and overall health care costs.

The program builds on several pillars: patient education to improve patient empowerment; patient-held mobile phone for daily data acquisition and transmission of blood pressure, heart rate, weight, well-being, and drug intake; physician-controlled telemonitoring of these data; nurse-led care for early detection of imminent decompensation; continuous optimization of guideline-based heart failure therapy for long-term stabilization; and finally, network communication to assure comprehensive heart failure management across venues.

The program was gradually developed, with all phases accompanied by program evaluation. First, between 2010 and 2015, shared decision making, responsibilities and liabilities of stakeholders, workflow and communication, information technology infrastructure, inclusion and exclusion criteria, duration and intensity, organizational integration, health care cost-effectiveness analysis, remuneration of stakeholders, and business models were implemented, evaluated, and continuously improved. A total of 137 patients were managed in these early phases of the HerzMobil Tirol program.

### Disease Management Processes in HerzMobil Tirol

**Figure 1:**
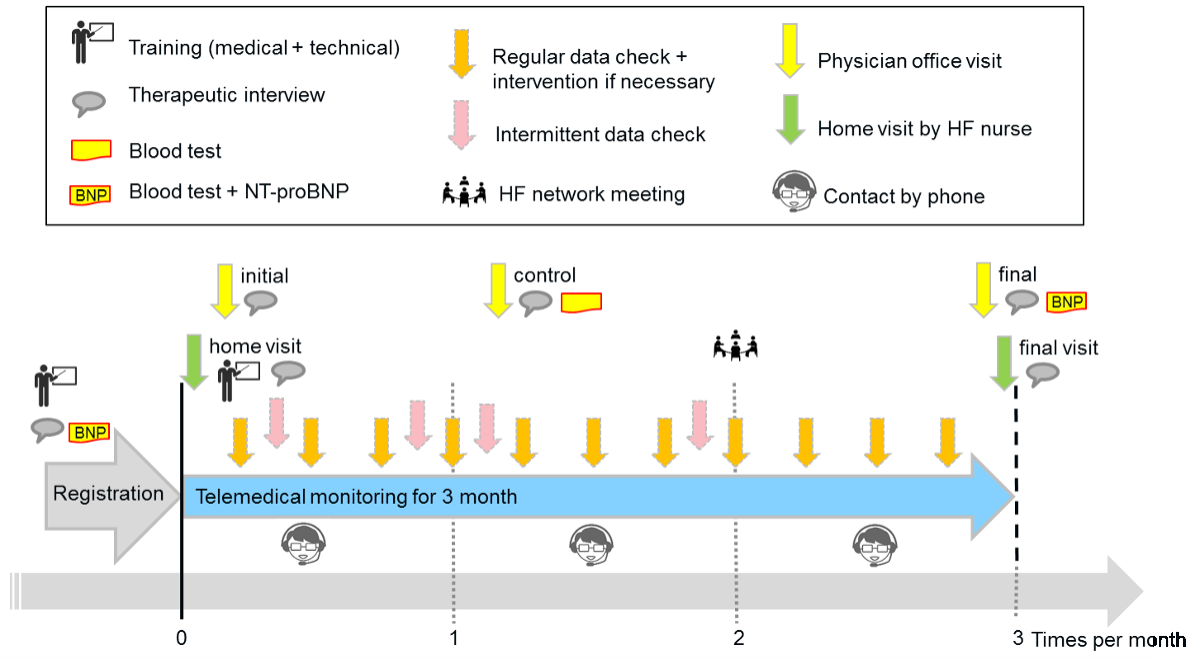
Integrated care process of HerzMobil Tirol. NT-proBNP: N-terminal prohormone of brain natriuretic peptide; HF: heart failure.

All participants of HerzMobil Tirol (physicians and nurses) communicate regularly to ensure optimal patient treatment without delay. Relevant information is shared on a Web-based telehealth software [18]. Figure 1 shows details of the process of patient management.

Heart failure patients enter the HerzMobil Tirol program after being hospitalized for acute heart failure. On discharge, each patient is assigned to one of the network physicians in private practice. This network physician supervises the heart failure management of the patient and optimizes the therapy. Discharge information from the hospital is communicated to the assigned network physician via the telehealth software.

Within HerzMobil Tirol, patients are supervised for 3 months. This period can be prolonged for another 3 months in case of heart failure instability. Over these 3 months, the network physician reviews telemedical patient data (blood pressure, heart rate, weight, well-being, and drug intake) and commentaries of the heart failure nurse at least once a week (regular data check). Out-of-limit data that are detected automatically by the telehealth system are highlighted and reviewed daily so that interventions, for example, adjustment of diuretics, can be taken immediately (intermittent data check).

Follow-up face-to-face visits of the patient with the network physician are scheduled 1, 4, and 12 weeks after discharge. Blood tests, for example, renal function tests, electrolytes, and N-terminal prohormone of brain natriuretic peptide, taken at hospital discharge and follow-up visits at the physician’s office allow for targeted optimization of medical therapy.

After entering the program, although the patient is still in the hospital, a heart failure nurse provides patient education both on the disease as well as on the mobile technology. During the 3 months of monitoring, these nurses are also responsible for compliance monitoring, phone contact with patients if required, and adjustments of medical therapy according to instructions of network physicians. To support this, a home visit by the heart failure nurse is scheduled immediately after discharge to finalize disease- and equipment-related education and to make sure that prescribed medication is available.

After 3 months, at the end of the managed care program, structured transfer of patients to regular care is organized.

Regular heart failure network meetings of all stakeholders (physicians and nurses) are scheduled every 3 months to support the exchange of experiences and the optimization of the technical and organizational network.

### Telemedical Technology in HerzMobil Tirol

HerzMobil Tirol uses an integrated concept called Keep-In-Touch (KIT) to facilitate efficient and reliable daily data documentation and transfer of blood pressure, heart rate, weight, well-being, and drug intake [19]. Every patient included in HerzMobil Tirol is provided a blood pressure and heart rate monitor and a weighing scale as well as a near-field communication–enabled mobile phone for daily data acquisition and transmission.

Heart failure nurses introduce patients to this equipment. Patients can call a helpdesk in case of technical problems. As most of the patients are elderly patients, the dialogue-oriented and process-supporting KIT technology and the mobile app are designed to support the patients at home in easy and secure handling of the daily data acquisition process [20].

To identify upcoming adverse events, signal processing algorithms are used to analyze the transmitted physiological data [20]. Automatic event detection that identifies both missing values as well as off-limit measurements indicates the need for immediate actions and fosters attention to those patients who might need early therapeutic intervention. The limits used for automatic event detection are individually defined and regularly adapted for each patient by the network physician.

**Figure 2:**
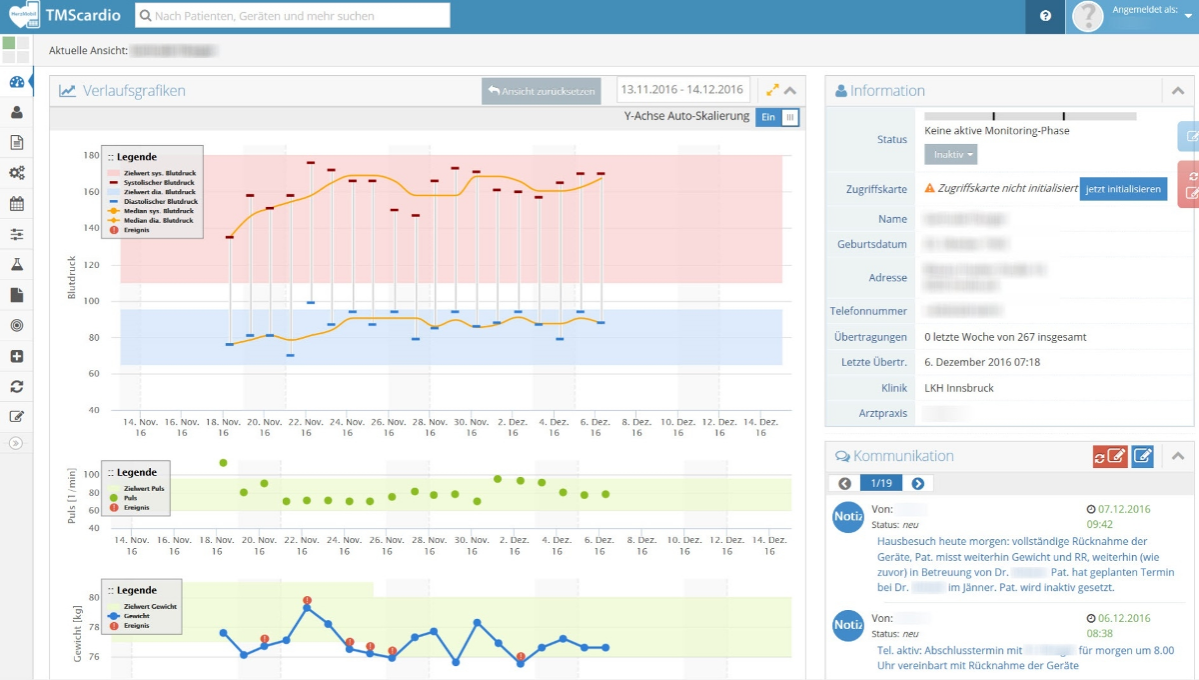
Stakeholder-specific dashboards of the Web-based telehealth system HerzMobil. Top chart: systolic (red) and diastolic (blue) blood pressure with daily measurement (−) and target range (red/blue area). Middle chart: heart rate with daily measurements (green dots) and target range (yellow area). Bottom chart: weight with daily measurements (blue dots) and target range (yellow area). Red exclamation marks: automatic alert on an event that has to be dealt with (eg, sudden change of weight or reaching predefined alerting values). Upper right: demographics of the patients (name, birth date, and address) as well as number of weekly data transmissions. Lower right: communication notes between physicians and nurses on this patient.

The Web-based telehealth software is made available to all stakeholders (network physicians, nurses, helpdesk, and network coordinator) and supports their individual tasks by user-specific dashboards (Figure 2). Each user can access the telehealth software using personal login information, normally via their computer or laptop. After login, a list of patients who are monitored by the user (eg, all patients being monitored by the network physician as user) is displayed. After selecting a patient, the monitoring interface, as shown in Figure 2, is shown.

The Web-based telehealth software is not integrated into the electronic health records of the various users because of the heterogeneity of the used software products (all hospitals and all network physicians are using electronic health records from different vendors).

### Data Collection

Ethical approval for the study was given by the ethical committee of the Medical University of Innsbruck.

During the prospective, pilot, single-arm study, the network comprised 16 physicians and 5 nurses. Patients hospitalized at the University Hospital of Innsbruck for acute heart failure during January and September 2016 were assigned to HerzMobil Tirol irrespective of the underlying heart disease. Patients with end-stage heart failure or relevant comorbidities (Charlson comorbidity score>5) associated with a life expectancy of less than 6 months or patients who could not use the provided devices were excluded from the program.

Patients were recruited during hospitalization and were surveyed at baseline as well as after 3 months using the following instruments:

- Kansas City Cardiomyopathy Questionnaire (KCCQ) is a validated 23-item survey to assess the health status and quality of life of patients with cardiomyopathy [21]. It addresses physical limitation, symptoms, self-efficacy, social limitations, and quality of life. A summary score and a clinical score are calculated. The scores range from 0 to 100, with higher scores indicating better health status.
- European Heart Failure Self-Care Behavior Scale (EHFScB-9) is a 9-item scale to assess self-care behavior of heart failure patients. It has been validated [22] and is available in German [23]. Overall self-care behavior is calculated by adding the scores of the 9 items, leading to scores from 9 to 45. A score of 9 indicates best self-care behavior.
- Information System Success Model Survey was developed based on the Delone and McLean Information System Success Model [24]. It consists of questions on the 6 dimensions: information quality (3 items), system quality (6 items), service quality (4 items), intention to use (7 items), user satisfaction (3 items), and net benefits (9 items). The instrument also contains 6 open questions on benefit and possibilities for improvements. The instrument was adapted from earlier studies, but is not formally validated.

In addition, the project team conducted 3 workshops with all network physicians and nurses to discuss the feasibility of HerzMobil and to derive lessons learned for future improvements of both organizational and technical components.

## Results

### Participants

A total of 50 patients were eligible and contacted between January and September 2016. From these patients, 43 patients agreed to join the HerzMobil Tirol program. Seven patients left the program because of incompliance or technical problems. Moreover, 1 patient died before completion. Of the remaining 35 patients, 28 completed all questionnaires. Mean age of these patients was 67 years (range: 43-86 years), 23 were male, and 5 were female (see Table 1).

The patients had between none and 6 comorbidities (median: 2), mostly history of myocardial infarction (7 patients), light liver disease (5 patients), and peripheral arterial occlusive disease (5 patients). A total of 15 of the 28 patients (15/28, 54%) had used computers before the study, and 16 patients (16/28, 57%) had used mobile phones.

### Kansas City Cardiomyopathy Questionnaire Health Status

**Figure 3:**
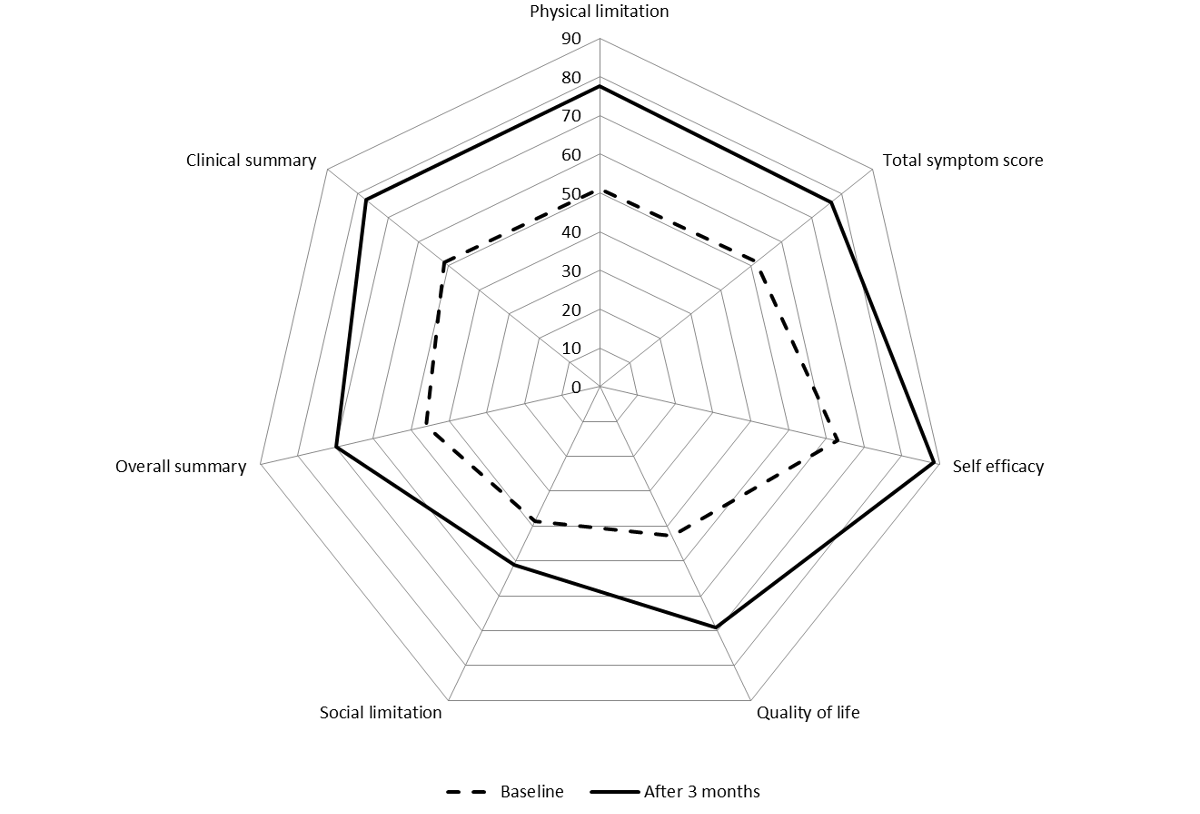
Change of health status (Kansas City Cardiomyopathy Questionnaire score; n=28; 0=minimum, 100=maximum) at baseline and after 3 months.

The overall summary KCCQ score (range: 0-100) increased from 46.2 at baseline to 69.8 after 3 months. The clinical summary KCCQ score increased from 51.4 to 77.3 (see Figure 3). Physical limitation increased from 51.1 to 77.5; self-efficacy increased from 63.0 to 88.4; quality of life increased from 42.9 to 69.1; social limitation increased from 38.5 to 51.1; and the total symptom score increased from 51.6 to 76.3. All changes were significant (*P*<.05) with the exception of social limitation.

**Table 1:** Number and age of participants.

Participants	All	Male	Female
Recruited for study, n (%)	50 (100)	37 (74)	13 (26)
Never beginner, n (%)	7 (100)	5 (71)	2 (29)
Dropped out or died during study, n (%)	8 (100)	5 (62)	3 (38)
Completed participation in study, n (%)	35 (100)	27 (77)	8 (23)
Mean age of participants in years (minimum-maximum)	67.1 (43-86)	65.6 (43-6)	71.5 (43-85)
Completed all questionnaires (return rate)	28 (80)	23 (85)	5 (63)

### European Heart Failure Self-Care Behavior Scale, Revised Into a 9-Item Scale, Self-Care Behavior

**Figure 4:**
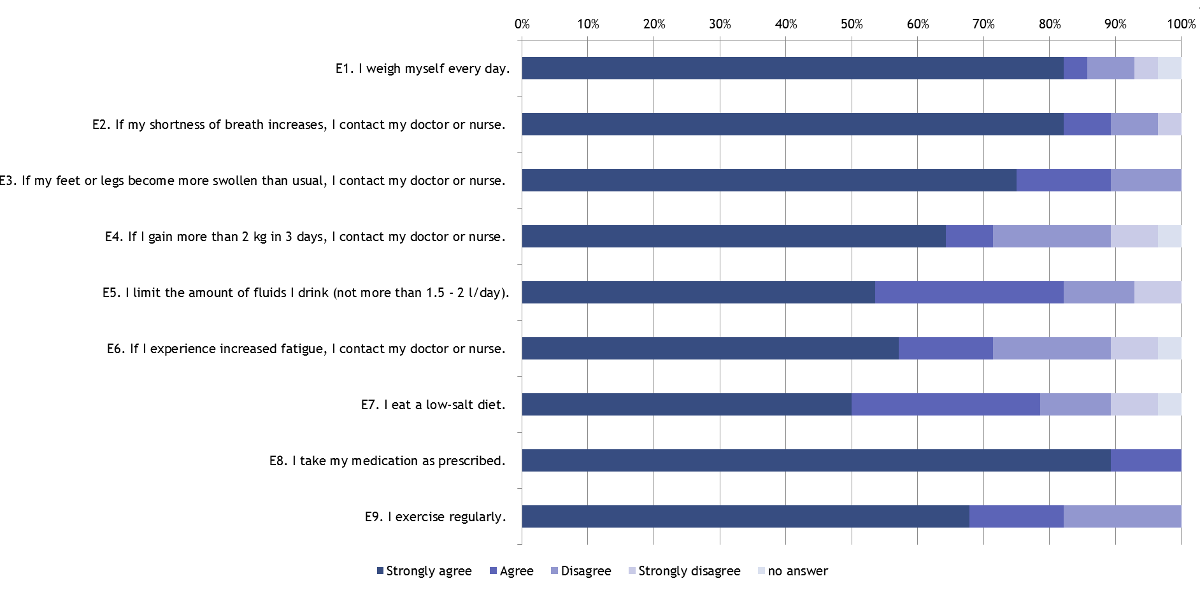
European Heart Failure Self-Care Behavior scale, revised into a 9-item scale, self-care behavior scores (n=29) after 3 months.

EHFScB-9 self-care behavior score after 3 months was 13.2 (SD 4.3), range was 9 to 22, with 9=best self-care behavior (see Figure 4).

### Patient Satisfaction

**Figure 5:**
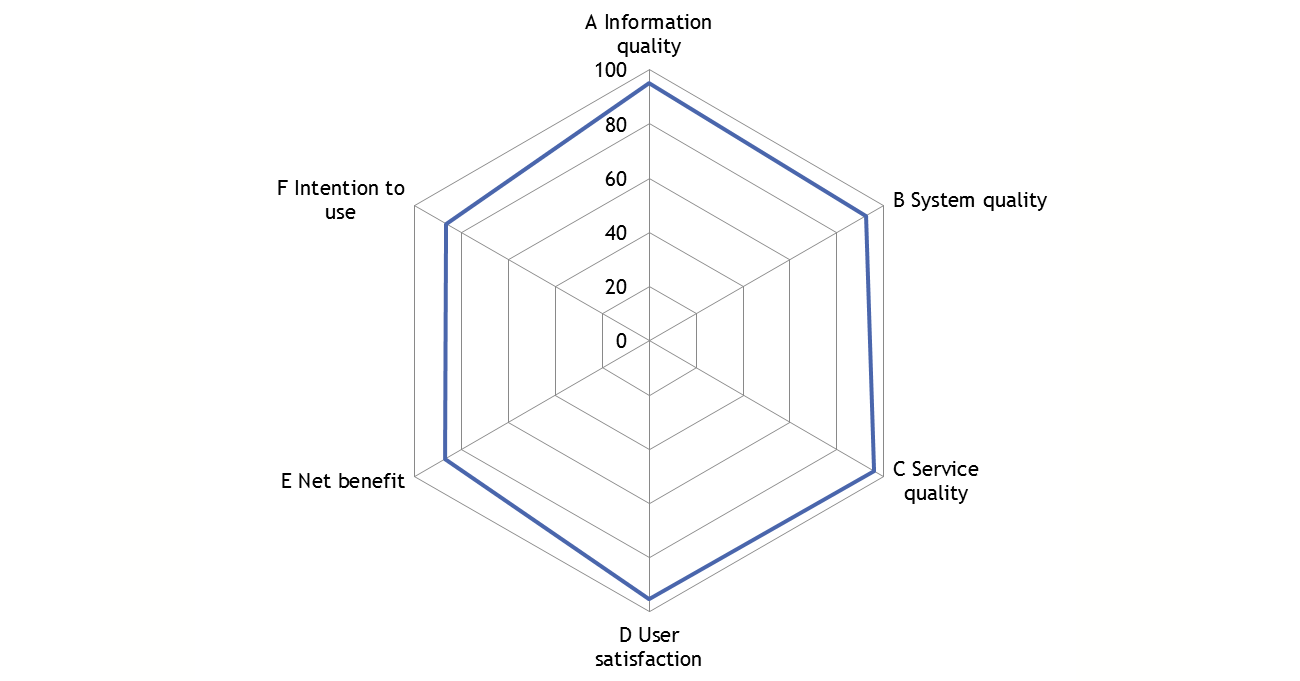
Aggregated results from the Information System Success Model survey (n=28) after 3 months (minimum=0%, maximum=100%). Information quality 95.2%; system quality 92.3%; service quality 95.8%; user satisfaction 95.2%; net benefit 87.1%; and intention to use 86%.

In all 6 dimensions of the Information System Success Model survey (range 0%-100%), mean results were 86% and higher (Figure 5), indicating high patient satisfaction.

**Figure 6:**
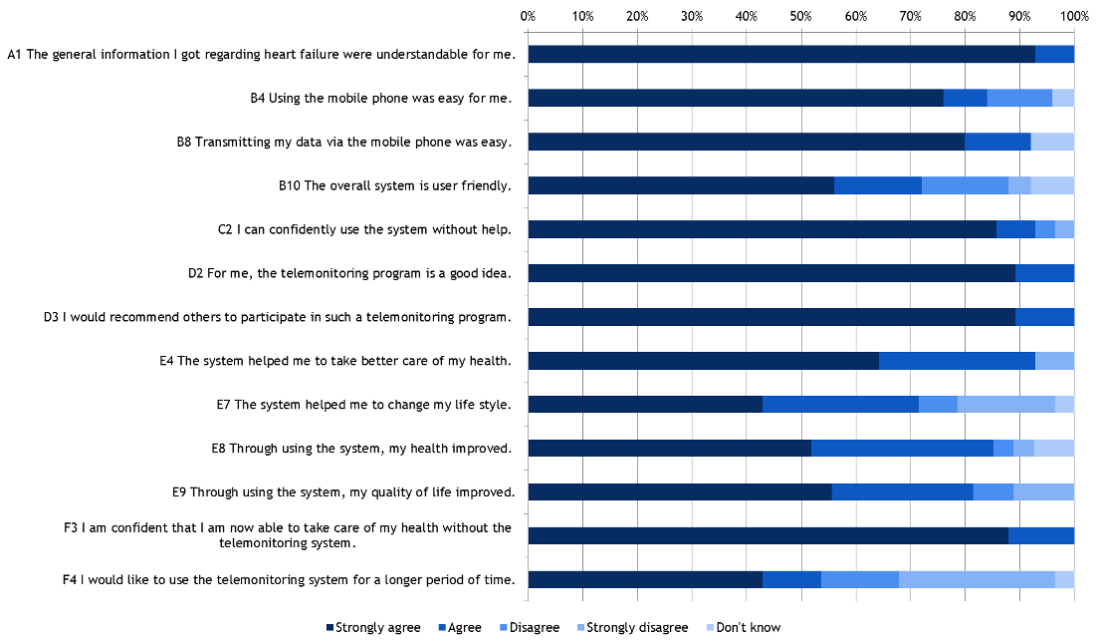
Selected items from the Information System Success Model survey (n=28) after 3 months.

A majority of patients (25/28, 89%) considered HerzMobil Tirol a good idea (D2, Figure 6) and would recommend it to others (D3). More than half of the patients (15/28, 53%) indicated that they would like to continue to use the telemonitoring system (F4). After 3 months, the majority of patients (22/28, 79%) indicated that they feel confident to be able to take care of their health without telemonitoring (F3).

In free text answers, 11 patients stated that they feel more secure because of the daily monitoring, and 4 patients stated that they are more confident in actively managing their disease. Moreover, 6 patients noted that the technology was somewhat unreliable and that the mobile phone was too complex for them.

## Discussion

Several studies already showed that home telemonitoring could reduce hospitalizations and mortality for patients with heart failure [25]. Here, we show our data on feasibility of home telemonitoring as part of a multidisciplinary postdischarge disease management program in everyday clinical practice. In addition, we will present lessons learned derived from this pilot study for further rollout of the program in the state of Tyrol.

### Summary of Study Results

Our results indicate that health status clearly improved in the most vulnerable phase of heart failure, as indicated by improved KCCQ and EHFScB-9 scores. Patients reported a high level of satisfaction with the quality of the system and the support and with the individual benefits of participation and showed good self-care behaviors after 3 months. In particular, the elderly patients seemed quite capable of using the mobile phone for data acquisition and data transmission. Only 5 patients (18%) stated that they received help from relatives. Thus, although age is often seen as a barrier to the use of technology [26], we found that this barrier can be addressed with patient education, good support by relatives, and a dedicated helpline. It must be noted, however, that 7 patients left the HerzMobil Tirol program because of in compliance or technical problems and were thus not included in data analysis.

### HerzMobil Tirol as a Disease Management Program

The transition between hospital and home after admission for acute heart failure is a vulnerable period marked by unplanned emergency room visits, hospital readmissions, and a high risk of death [8,9]. The importance of effective communication between health care professionals involved with inpatient and community care [27], intense patient education to improve self-empowerment, constant medication reconciliation and therapy optimization according to prevailing guidelines, structured outpatient follow-up for early indicators of clinical decompensation for a seamless transition from the hospital to community, and the central role of specialized heart failure nurses in this complex interplay is well recognized [11,27-29]. A reliable and stable telemedical network is the facilitator for such an organizational network.

HerzMobil Tirol is designed as such a multidisciplinary postdischarge disease management program for heart failure patients using a telemedical monitoring system incorporated in a comprehensive network of specialized heart failure nurses, nonhospital-based physicians, and referral centers. The telemonitoring system HerzMobil Tirol relays physiological data, information on patient’s symptoms, and drug adherence for review to the health care professionals. The gradual development of the program finally resulted in the decision to offer a concentrated 3-month health care service covering the most vulnerable phase after discharge.

Nonhospital-based network physicians and heart failure nurses play a central role in this network program. Shared decision making and effective communication between heart failure nurses and network physicians are based on the well-defined workflow and responsibilities. Intense education of patient and their families within and outside the hospital by trained heart failure nurses and structured follow-up by network physicians are central for sustained disease stabilization. Meticulous pre- and postcare evaluation of patients ensures quality control, thus continuous optimization of the program.

### Comparison With Other Studies

**Figure 7:**
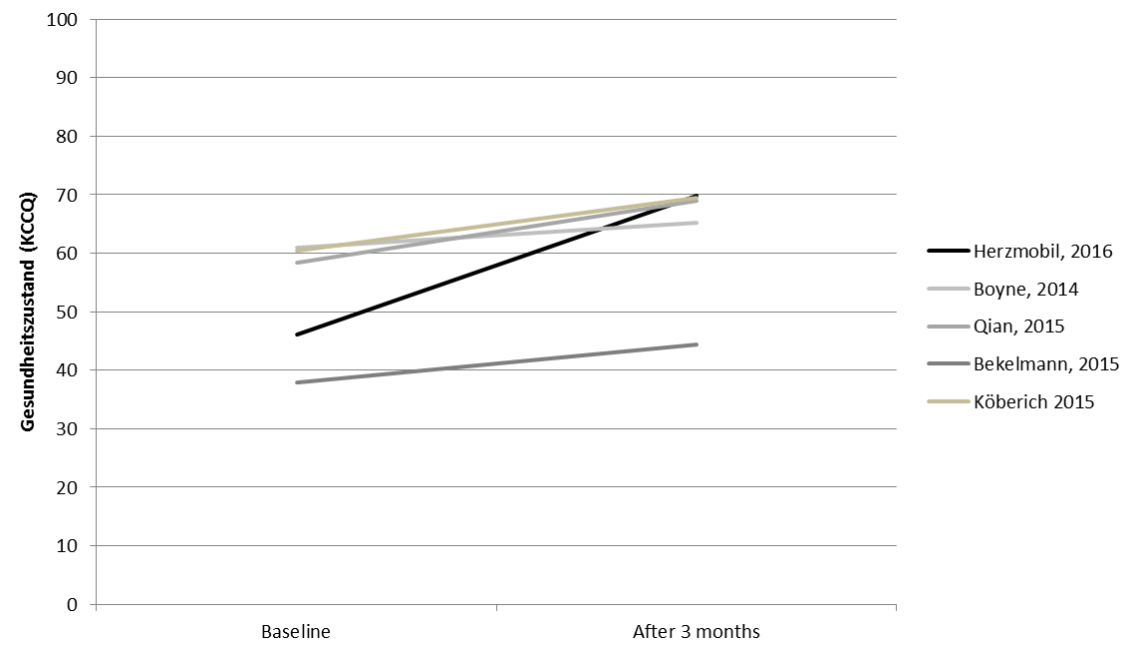
Change of health status (Kansas City Cardiomyopathy Questionnaire, KCCQ score) in HerzMobil Tirol at baseline and after 3 months in comparison with 4 other studies.

Health status of patients at discharge in HerzMobil Tirol measured by the KCCQ score was lower than that in other studies, indicating better health status. Improvement in the KCCQ score after 3 months was higher than that in other studies (Figure 7): Boyne et al measured KCCQ for 382 patients with stable heart failure who received 6 months of daily telemonitoring, combined with 2 hospital check visits [30]. Quian et al measured KCCQ for 1427 patients with recent inpatient treatment for heart failure who received 6 months of daily telemonitoring [31]. Bekelmann et al [32] measured KCCQ for 392 patients with known heart failure who participated in a collaborative heart failure network combined with 6 months of telemonitoring. Köberich et al [33] measured KCCQ for 122 patients who were hospitalized due to heart failure, received special training, and then a 3-month phone-based monitoring. Although the studies of Boyne and Bekelmann included patients with stable heart failure, Qian and Köberich included patients with unstable heart failure, which is comparable with HerzMobil Tirol. Still, patients in HerzMobil Tirol showed the strongest improvement of the KCCQ score (Figure 7).

Self-care behavior measured by the EHFScB-9 after 3 months of care (mean: 13.2) indicated better self-care behavior compared with the following studies: Lee et al reported a mean EHFScB-9 score of 18.1 for 200 heart failure patients [22]. Koeberich et al found a mean EHFScB-9 score of 19.9 for 109 heart failure patients [23]. The same group showed a mean EHFScB-9 score of 19.6 at baseline and of 16.5 after 3 months for a group of 58 discharged heart failure patients who received special training, combined with telephone support over 3 months [33]. Comparatively, the mean EHFScB-9 score in HerzMobil Tirol patients of care was lower.

### Limitations of the Study

The study was designed as a pilot study without a control group. Hence, analyses are limited to pre- and postcomparisons and comparison with published data. It cannot be entirely excluded that improvement in health status after 3 months is part of a gradual stabilization after hospitalization. Yet, comparison with published data from other studies suggests a better outcome of patients in HerzMobil Tirol with regard to health status and self-care behavior.

Self-care behaviors were measured only at the end of the third month. To address this limitation, self-care behaviors of HerzMobil Tirol patients were also compared with data from other studies.

Outcome was measured using survey instruments. As of now, no long-term evaluation of rehospitalization and other adverse events is available for HerzMobil Tirol.

### Implications and Recommendations for Practice

Telemonitoring for heart failure patients is not new, and several studies have established the feasibility of these approaches and its positive impact on clinical outcome [34-36]. However, HerzMobil Tirol is a telemonitoring project that was able to transform from a pilot study to a routine care project. On the basis of the experiences in transferring from a pilot to routine care, several lessons can be derived. They were developed in repeated workshops with network physicians and network nurses and may be useful for other disease management programs for heart failure. These recommendations are also supported by a recent position paper on disease management of heart failure of the Austrian Society of Cardiology that also stresses these aspects [17].

First, it is crucial to decide on the temporal positioning of managed care in the trajectory of heart failure. In the vulnerable period that is directly after discharge, focus in HerzMobil Tirol is on the seamless transition from the hospital to the telemedical network and on a 3-month monitoring. Effective communication between health care providers in various sectors is mandatory in this phase. This requires a well-organized telemedical platform that can be easily and safely accessed by all stakeholders. In contrast, in programs supporting the chronic phase, the length of telemonitoring is often not clearly defined, and communication between sectors of care, in general, is less challenging here.

Second, in the vulnerable period, patient education and constant optimization of disease-modifying therapy are particularly important. Defining the predominant gaps in health care in a region and building HerzMobil Tirol on existing health care infrastructures are essential for a high acceptance by payers and caregivers.

Third, a solid network structure and well-defined processes are more important than the specific telemonitoring tools that are used. For example, remote follow-up either by telephone or by telemonitoring must be made available in a well-functioning network of health care professionals to ensure constant review and timely response.

Fourth, the implementation process must be clear and transparent and avoid work overload, particularly to the heart failure nurses. A dedicated program coordinator is helpful to orchestrate all stakeholders and manage efficient cooperation of all partners. This strengthens the acceptance by health care professionals. In addition, legal aspects and adequate remuneration of stakeholders have to be settled.

Fifth, because of the central role of heart failure nurses, providing specialized training for nurses is a mandatory prerequisite before commencing such a program. In addition, meticulous introduction of all stakeholders into the processes and clear definition of the particular role as well as constant training for the entire personnel has to be organized.

Sixth, the first months after commencing a program are mostly dedicated to the optimization of organizational issues. Hence, it is helpful to avoid inclusion of multimorbid patients in this initial phase to not push the boundaries of the program by too complex interventions.

Summarizing, to establish a multidisciplinary postdischarge disease management network for heart failure patients, a large number of organizational, technical, legal, and economic questions have to be answered and tested in pilot projects before such a program can be transferred from a project status into routine care. In the case of HerzMobil Tirol, this process took 6 years overall.

### Conclusions

The steady increase in the number of chronic patients due to the demographic trend and the ongoing medical progress shows the weaknesses of the Austrian health care system. A historically grown separation between in-patient and out-patient care leads to an insufficient consideration of the medical needs of patients along with their carers.

Telehealth care, as HerzMobil Tirol, can to some extent solve this problem and should be seen as a strategical investment to improve medical care. The telemedical information and communication technologies used in HerzMobil Tirol can help to overcome organizational and sectoral boundaries.

The role and competencies of the involved health professionals also have to be defined. HerzMobil Tirol has highlighted, for example, that specialized heart failure nurses will gain importance. Especially for Austria, which has a lot of potential for improvements in this field, this is an important insight.

Telehealth care is far more than a technology project. Instead, it enables lasting innovations for the health care system. HerzMobil Tirol showed that telehealth care is a trigger for change and innovation in the health care system. This is an important finding, especially in complex and change-resistant systems such as the health care system.

HerzMobil Tirol was officially introduced in the province of Tyrol in July 2017. This is the first telehealth care program in Austria regularly financed by health insurances. On the basis of the positive experiences, the province of Tyrol is also willing to establish further telehealth care projects in other relevant indications. The rollout in Tyrol will be done gradually. At the moment, specific regions are selected where more HerzMobil Tirol networks of hospital(s), physicians, and nurses are being established. Experiences from the already running regions are made available to the new regions to facilitate fast rollout. Further evaluation of long-term impact of HerzMobil Tirol is planned. Experiences of HerzMobil Tirol are also made available to other Austrian federal states, yet Austrian-wide rollout is dependent on political and financial decisions that are outside the control of the project team.

## Abbreviations

EHFScB9: European Heart Failure Self-Care Behavior Scale
KCCQ: Kansas City Cardiomyopathy Questionnaire
KIT: Keep-In-Touch
